# Prediction of Mortality in Patients After Oncologic Gastrointestinal Surgery: Comparison of the ASA, APACHE II, and POSSUM Scoring Systems

**DOI:** 10.7759/cureus.13684

**Published:** 2021-03-04

**Authors:** Nagihan Gozde KISA, Emre KISA, Banu Eler Cevik

**Affiliations:** 1 Anesthesiology and Reanimation, Golcuk Necati Celik State Hospital, Kocaeli, TUR; 2 Anesthesiology and Critical Care, Derince Education and Research Hospital, Kocaeli, TUR; 3 Anesthesiology and Reanimation, Kartal Dr. Lutfi Kirdar Research& Education Hospital, Istanbul, TUR

**Keywords:** icu mortality rate, possum, asa, apache ii, gastrointestinal cancer surgery, morbidity and mortality

## Abstract

Scoring systems have been developed to predict the expected mortality and morbidity in surgical procedures. In this study, our aim was to compare the ASA (American Society of Anesthesiologists), APACHE (Acute Physiology and Chronic Health Evaluation) II, POSSUM (Physiological and Operative Severity Score for the enUmeration of Mortality and morbidity) scoring systems as predictors of mortality in patients who underwent gastrointestinal oncologic surgery, followed, and were admitted to the intensive care unit during the postoperative period. We examined the files of 82 patients who underwent oncologic gastrointestinal surgery and followed up in the intensive care units (ICUs). The patients’ APACHE II scores and predicted mortality rates (PMR) according to the APACHE II, POSSUM, and ASA scores were ​​calculated. The receiver operator characteristic (ROC) curve analysis was used when evaluating the performances of the ASA, APACHE, and POSSUM scoring systems in terms of accurate assessment of mortality. Accordingly, the area under the curve (AUC) = 0.5 no distinction, 0.5 <AUC <0.7 discriminative power of the test is statistically not significant, 0.7 <AUC <0.8 acceptable, 0.8 <AUC <0.9 very good and 0.9 <AUC <1 perfect. The evaluations showed that APACHE II had the best performance with 0.81, followed by POSSUM, which had an acceptable level at 0.78. On the other hand, the ASA score was 0.63 and its discriminative power was identified as statistically insignificant. Our results show that the POSSUM and APACHE II scoring systems were better at predicting mortality than the ASA scoring system for the prediction of mortality in the postoperative period. Both the POSSUM and APACHE II scoring systems can be confidently used for the prediction of mortality in patients undergoing operations due to oncologic gastrointestinal diseases.

## Introduction

Surgeries for gastrointestinal tumors are frequent oncologic surgical cases, and both genetic and environmental factors have been known to play important roles in the etiology [1]. Scoring systems have been developed to determine the mortality and morbidity in these surgical procedures. The ASA (American Society of Anesthesiologists) scoring system is the preoperative classification used to estimate peri/postoperative and mortality in patients who will undergo surgery; it is a simple, subjective measure of comorbidity that has been widely used since 1963 [2]. This scoring system is a reliable indicator for risk, and its predictive value increases when age is taken into account [3].

The APACHE (Acute Physiology and Chronic Health Evaluation) score was used for the first time in 1981. The sum of the scores between 0 and 4, which is a specified index for 34 physiological measurements concerning seven major organ systems, forms an acute physiology score. The APACHE scoring system has also been found to be useful for comparing the success of the intensive care unit (ICU). However, this system is too complex for routine use; therefore, Knaus et al. developed the APACHE II system, which is simpler and clinically more useful [4].

A more physiological scoring system is the POSSUM (Physiological and Operative Severity Score for the enUmeration of Mortality and morbidity) scoring system, which was developed between 1991 and 1998 and is still used today. The POSSUM uses 12 physiological variables to give a physiological score and a six-factor operative severity score to give an operative mortality score. The parameters of the physiological score are age, Glasgow score, respiratory function, urea, heart rate, cardiac failure, hemoglobin, leukocyte count, electrocardiogram (ECG) changes, sodium, potassium, and systolic blood pressure. At the same time, using these parameters, POSSUM also determines the postoperative morbidity score. The POSSUM scoring system determines the preoperative mortality risk with more qualitative data [5-7]. Over time, POSSUM has become a scoring system that can be used in the estimation of the outcomes that may occur following vascular, upper gastrointestinal, pulmonary, and colorectal surgeries [8].

In this study, our aim was to compare the ASA, APACHE II, POSSUM scoring systems as predictors of mortality in patients who underwent gastrointestinal oncologic surgery and were admitted to the ICU during the postoperative period.

## Materials and methods

In this retrospective study, we examined files of patients who underwent oncologic gastrointestinal surgery and followed up in the ICUs. Ethics committee approval was not required because anonymous data were used. All data were collected retrospectively. We accessed patient files through archives and our hospital’s information processing systems. Patients diagnosed with esophageal, stomach, pancreatic, and bowel cancers were included in the study. Age, gender, ASA score, APACHE II score, the predicted mortality rates (PMR) according to the APACHE II and POSSUM score, type of operation, associated diseases (chronic obstructive pulmonary disease, coronary artery disease, congestive heart failure, hypertension, and diabetes mellitus), and mortality were recorded in the prepared forms.

Operation types were divided into two groups as follows: major operations (any kind of laparotomy, colon resection, cholecystectomy, and choledochal exploration) and major complex operations (abdominoperineal operations, pancreas and liver resection, and esophageal and gastric resection).

Morbidity was defined as any complication occurring within the first 30 postoperative days. Local complications were those that arose on the surgical wound without systemic repercussions. Systemic complications were defined as anything that affected the normal physiology such as pneumonia, respiratory failure, sepsis, cerebral vascular accident, acute myocardial infarction, massive bleeding requiring reoperation, and operative death. Any death within 30 days of operation was classified as an operative death.

In our retrospective study, the patients’ APACHE II scores and PMRs according to APACHE II were ​​calculated electronically using the www.kalite.saglik.gov.tr website that belongs to the Turkish Ministry of Health. Meanwhile, the POSSUM scores were also calculated electronically using the www.sfar.org and www.riskprediction.org.uk websites. Obtained mortality rates were recorded.

Statistical analysis of our study’s data was performed using the Statistical Package for the Social Sciences (SPSS) software for Windows 13.0. When evaluating the descriptive statistical parameters (mean, standard deviation, frequency), the comparison between two groups was done by the chi-square test, student's t-test, and the Mann-Whitney U test. The Shapiro-Wilk test was used for normality of the data while multiple comparisons were done using one-way analysis of variance (ANOVA). Significance was set at p <0.05. The receiver operator characteristic (ROC) curve analysis was used when evaluating the performances of the ASA, APACHE, and POSSUM scoring systems in terms of accurate assessment of mortality. Accordingly, area under the curve (AUC) = 0.5 no distinction, 0.5 <AUC <0.7 discriminative power of the test is statistically not significant, 0.7 <AUC <0.8 acceptable, 0.8 <AUC <0.9 very good, and 0.9 <AUC <1 perfect.

## Results

The mean age of the patients included in our study was 64.6 years (24-84), and the female:male ratio was 36:46. The diagnostic distribution of patients was as follows: 16 patients with pancreatic cancer, seven with esophagus cancer, 26 with gastric cancer, 18 with colon cancer, and 15 with rectal cancer.

When we evaluated the relationship between age and mortality rate, we determined the following: four of the patients < 70 years group (4.8%) and five for the ≥ 70 years group (17.5%) were dead. The mortality rate was found to increase with increasing age; however, this increase was not statistically significant.

Fifty-three of our patients (64%) underwent major complex surgery while 29 (36%) underwent major surgery. The mortality rate of patients undergoing major surgery was 13.8% while the mortality rate of patients undergoing major complex surgery was 9.4%. There was not a statistically significant relationship between mortality and the type of surgery performed.

The ASA scoring was classified into three classes: II, III, and IV, where the mortality rate of ASA class IV exceeded the mortality rates of other classes almost threefold. However, the statistical evaluation showed that there was no significant difference between ASA classes.

The APACHE II scores were divided into three groups (≤20, 20-25, ≥25). The group that had APACHE II scores ≤20 had 13 patients, and no mortality was observed. Next, the group that had scores between 20 and 25 had 24 patients and no mortality was observed. On the other hand, the group that had APACHE II scores ≥25 had 45 patients and a mortality rate of 20%. The statistical analysis determined the p-value to be 0.015 and showed that there was a significant relationship between APACHE II scoring and mortality. In addition, PMR increased in parallel with APACHE II scores, and the relationship between PMR and mortality was statistically significant.

The POSSUM scores were evaluated under five different groups (≤10, 11-20, 21-30, 31-40, and ≥41). The group that had POSSUM scores ≤10 had 17 patients and no mortality. The group with scores between 11 and 20 had 36 patients, and their mortality was found to be 5.6%. Moreover, there were 13 patients in the group with scores between 21 and 30, and their mortality rate was 15.4% while the group that had scores between 31 and 40 had nine patients and their mortality rate was identified as 22.2%. Lastly, seven patients were in the group with scores ≥41, and this group had the highest mortality rate at 42.9%. It was determined that in these patients, an increase in the POSSUM score correlated with the increase in mortality rate. The statistical analysis showed the p-value to be 0.016 and determined that the relationship between POSSUM scoring and mortality was statistically significant (Table 1).

**Table 1:** Descriptive table ASA: The American Society of Anesthesiologists, APACHE: Acute Physiology and Chronic Health Evaluation, POSSUM: Physiological and Operative Severity Score for the enUmeration of Mortality and morbidity

Classification	Groups	Number of patients	Survival	Non-survival	p:
Age	<70	42	40	2	0.0984
	≥70	40	33	7	
Gender	Female	36	31	5	0.5048
	Male	46	42	4	
Operation type	Major	29	26	3	0.9035
	Major Complex	53	47	6	
Cancer type	Esophagus	7	5	2	0.384
	Gastrum	26	24	2	
	Pancreas	16	13	3	
	Colon	18	16	2	
	Rectum	15	15	0	
ASA	II	15	14	1	0.2829
	III	56	51	5	
	IV	11	8	3	
APACHE II	<20	13	13	0	0.015
	20-25	24	24	0	
	≥ 25	45	36	9	
POSSUM	≤ 10	17	17	0	0.016
	11-20	36	34	2	
	21-30	13	11	2	
	31-40	9	7	2	
	≥41	7	4	3	

**Figure 1:**
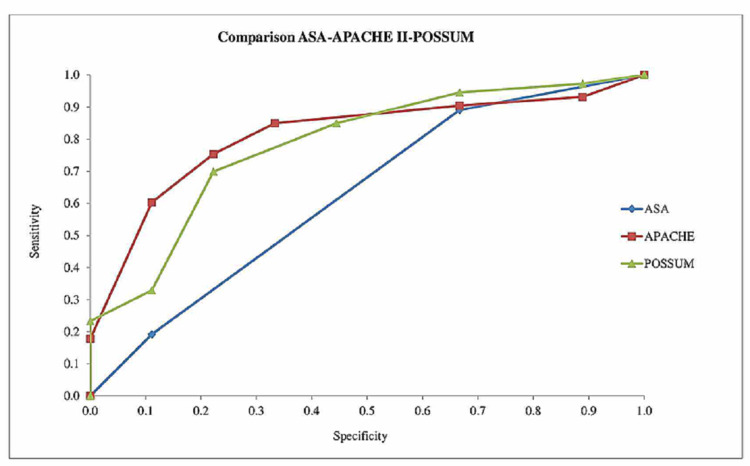
Comparison of the ASA-APACHE II-POSSUM scoring systems ASA: The American Society of Anesthesiologists, APACHE: Acute Physiology and Chronic Health Evaluation, POSSUM: Physiological and Operative Severity Score for the enUmeration of Mortality and morbidity

The evaluations showed that APACHE II had the best performance with 0.81, followed by POSSUM, which had an acceptable level at 0.78. On the other hand, the ASA score was 0.63 and its discriminative power was identified as statistically insignificant (Table 2, Figure 1).

**Table 2:** Comparison of ASA-APACHE II-POSSUM scores AUC=0.5 no distinction, 0.5<AUC<0.7 statistically not significant, 0.7<AUC<0.8 acceptable, 0.8<AUC<0.9 very good and 0.9<AUC<1 perfect. ASA: The American Society of Anesthesiologists, APACHE: Acute Physiology and Chronic Health Evaluation, POSSUM: Physiological and Operative Severity Score for the enUmeration of Mortality and morbidity, ROC: receiver operator characteristic, AUC: area under the curve

	Area Under the ROC Curve=AUC
ASA	0.63
APACHE II	0.81
POSSUM	0.78

## Discussion

Physiological scoring systems have been developed with objectives such as diagnosing patients admitted to the ICU, clinical observation and data recording, identification of mortality and morbidity probabilities, the possibility of comparison between patients or between different intensive care units, and monitoring the quality of applications. Owing to these characteristics, these systems can serve as guides for the observation and treatment of patients. We found that the APACHE II and POSSUM scoring systems are demonstrative for predicting mortality rate gastrointestinal tract surgery. However, the ASA scoring system is not a good choice.

In the present study, we investigated patients who underwent surgery due to oncologic gastrointestinal diseases and determined that age, type of operation, number of associated comorbid diseases, and ASA classification alone are inadequate for prediction of mortality during the postoperative period. We suggest that it is much more appropriate to consider all these parameters as a whole for a much more accurate mortality prediction. Therefore, we report that the POSSUM scoring system, which is calculated by using physiological and operative parameters, and the APACHE II scoring system, which evaluates many physiological parameters together, are better at predicting mortality than ASA in the postoperative period (APACHE II mortality relationship: p = 0.015, POSSUM mortality relationship: p = 0.016). Törer calculated the POSSUM scores in 747 patients who underwent general surgery and compared their mortality and morbidity. The mean POSSUM score of patients was 15.8 ± 6.2. ROC was used to determine the relationship between morbidity and mortality rates based on the calculated POSSUM scores of patients. For mortality, the area under the curve was 0.746 ± 0.098 (p =0.016) while for morbidity, it was 0.626 ± 0.037 (p=0.003) [9].

In our studied patients, an APACHE II score of <25 was seen as no mortality as compared to a score of ≥25, which was associated with mortality of all nine patients. At a cut-off value of 25, the AUC (95%CI) was 0.81 (0.761-1.000). While different studies have so far shown the ability of APACHE II scores to predict mortality and similar AUC has been reported in other studies for patients calculating APACHE II for varied causes, as our study has demonstrated the strongest correlation to date with an AUC of 0.81 as compared to 0.65-0.86 in other studies [10-14].

The POSSUM score was calculated in various papers and an AUC value between 0.68-0.95 was figured out [10,13,15-18]. In our study, we found a value of 0.78 for the POSSUM scoring system, and this result was as remarkable as the APACHE II scores result.

Some of the studies recommended that the POSSUM scoring system was better to predict mortality than the APACHE II scoring system [10,13,19]. We found in our study that APACHE II is more suitable than the POSSUM score in predicting mortality, similar to some studies’ results [16]. On the other hand, the ASA classification system was insufficient with 0.63, similar to Cuijpers’ 108-patient series [20].

In our paper, we divided the POSSUM scores into five subgroups (≤10, 11-20, 21-30, 31-40, and ≥41). Our aim was to reach more refined and suitable mortality rates among subgroups in this division. Our study showed that a higher POSSUM score matched with higher mortality and was also statically eloquent. Karan et al. stated that the POSSUM score was good at predicting morbidity [21]. However, they were not able to check the usefulness of the scores for mortality because no death occurred. Bollschweiler et al. evaluated the POSSUM scoring system in 137 patients who underwent gastrectomy due to gastric cancer [17]. In their study, they divided patients into three groups: patients with low risk, medium risk, and high risk. The mortality rates of the low-risk group calculated by the POSSUM scoring system were between 0% and 20% while the actual mortality rate was 14%. The mortality risk in the moderate risk group was between 21 and 40 while the actual mortality rate was 23%. Meanwhile, the mortality rates in the high-risk group were 40 and above while the actual mortality was 50%. These values ​​were statistically significant. The authors concluded that the POSSUM scoring system could be useful in both perioperative and postoperative assessment. However, the studies reported that there was no other scoring system that evaluates and compares preoperative values with the postoperative course. Elias et al. evaluated the POSSUM scoring system’s prediction of mortality in 416 surgical patients [14]. They divided POSSUM scores into four groups, and they showed the same correlation between mortality and risk groups.

The smaller sample size limits the wider application of the results of this study. More research is required using bigger sample size and having longer follow-up periods to assess long-term complications. We could not reach some patients' 30-day trial conclusions because of not keeping in contact with the same hospital. 

## Conclusions

In conclusion, we suggest that the APACHE II and POSSUM scoring systems are more reliable in predicting mortality than the ASA scoring system in patients undergoing oncologic gastrointestinal surgery. Maybe the APACHE II score is more useful and practical than the POSSUM scoring system because of the simple calculations. On the other hand, these results may be crosschecked with broad participant meta-analyses.
